# Piezoelectric-based bioactive zinc oxide-cellulose acetate electrospun mats for efficient wound healing: an *in vitro* insight

**DOI:** 10.3389/fimmu.2023.1245343

**Published:** 2023-09-27

**Authors:** Sumanta Ghosh, Sumedh Vaidya, Namdev More, Ravichandiran Velyutham, Govinda Kapusetti

**Affiliations:** ^1^ Department of Medical Devices, National Institute of Pharmaceutical Education and Research- Kolkata, Chunilal Bhawan, Kolkata, India; ^2^ Department of Medical Devices, National Institute of Pharmaceutical Education and Research- Ahmedabad, Opposite Air Force Station, Gandhinagar, Gujarat, India

**Keywords:** nanofiber, bioactive, electrospinning, ZnO, cellulose, piezoelectricity, wound healing

## Abstract

Being a complex physiological process involving the removal of damaged tissue debris and creating a new microenvironment for host tissue regeneration, wound healing is still a major challenge for healthcare professionals. Disruption of this process can lead to tissue inflammation, pathogenic infections, and scar formation. Current wound healing treatments primarily focus on passive tissue healing, lacking active engagement in the healing process. In recent years, a new class of functional biomaterials based on piezoelectric properties has emerged, which can actively participate in the wound healing process by harnessing mechanical forces generated from body movement. Herein, we have fabricated a bioactive Cellulose Acetate (CA) electrospun nanofibrous mat incorporating zinc oxide (ZnO) and investigated its efficiency for accelerated wound healing. We have characterized the physicochemical properties of the fabricated nanofibrous mats using various assays, including SEM, FTIR, TGA, mechanical testing, degradation analysis, porosity measurement, hemolysis assay, and piezoelectric d_33_ coefficient measurement. Through our investigation, we discovered the tunned piezoelectric coefficient of fabricated specimens due to incorporating ZnO into the CA fibers. *In vitro* studies also confirmed enhanced cell adhesion, proliferation, and migration, indicating faster wound healing potential. Overall, our findings support the efficacy of piezoelectric-based ZnO-incorporated bioactive CA nanofibrous mats for efficient wound healing.

## 1 Introduction

In recent years, significant advancements have been made in developing bioactive wound dressing materials to improve efficiency (1, 2). While a wide range of wound dressing materials is available, from simple sutures to complex cell-loaded hydrogels, most primarily facilitate passive wound healing processes, lacking active engagement in endogenous cellular crosstalk (3). Thus, there is a growing need for bioactive dressing materials that possess the advantages of conventional dressings and actively participate in the tissue healing process. The physiological function of wound healing is mainly characterized by four overlapping stages: homeostasis, tissue inflammation, cellular proliferation, and tissue remodeling (4, 5). Following an injury, platelets accumulate at the wound site, initiating clotting and providing a platform for recruiting other infiltrating cells. Circulating monocytes transform into pro-inflammatory macrophages and secrete pro-inflammatory factors such as prostaglandins and cytokines, leading to an inflammatory response. During the proliferation and remodeling stage, keratinocytes and fibroblasts migrate to the wound site to restore normal skin function. Fibroblasts proliferate and differentiate into myofibroblasts, and collagen remodeling occurs, ultimately restoring dermal function (6, 7).

Cellulose, a well-explored biopolymer, possesses ideal characteristics for wound healing applications, including biocompatibility, biodegradability, excellent mechanical properties, affordability, high thermal stability, and remarkable water adsorption capability (8, 9). Cellulose acetate (CA), a widely used cellulose derivative, exhibits good electro-spinnability properties (10, 11). Moreover, the acetylation of cellulose imparts a unique hydrophobic characteristic that promotes protein and cell adhesion on electrospun scaffolds. The hydrophobicity of cellulose acetate is directly proportional to the degree of acetylation (12). Even though several researchers have employed cellulose acetate nanofibrous scaffolds as wound dressing materials, limitations remain. The pristine cellulose acetate does not actively engage with the typical healing phases, making it less pronounced for developing a bioactive scaffold. To overcome this limitation, incorporating secondary substances such as drugs, metal nanoparticles, or antibiotics during the electrospinning process is a common and established approach (13, 14).

**Figure 1:**
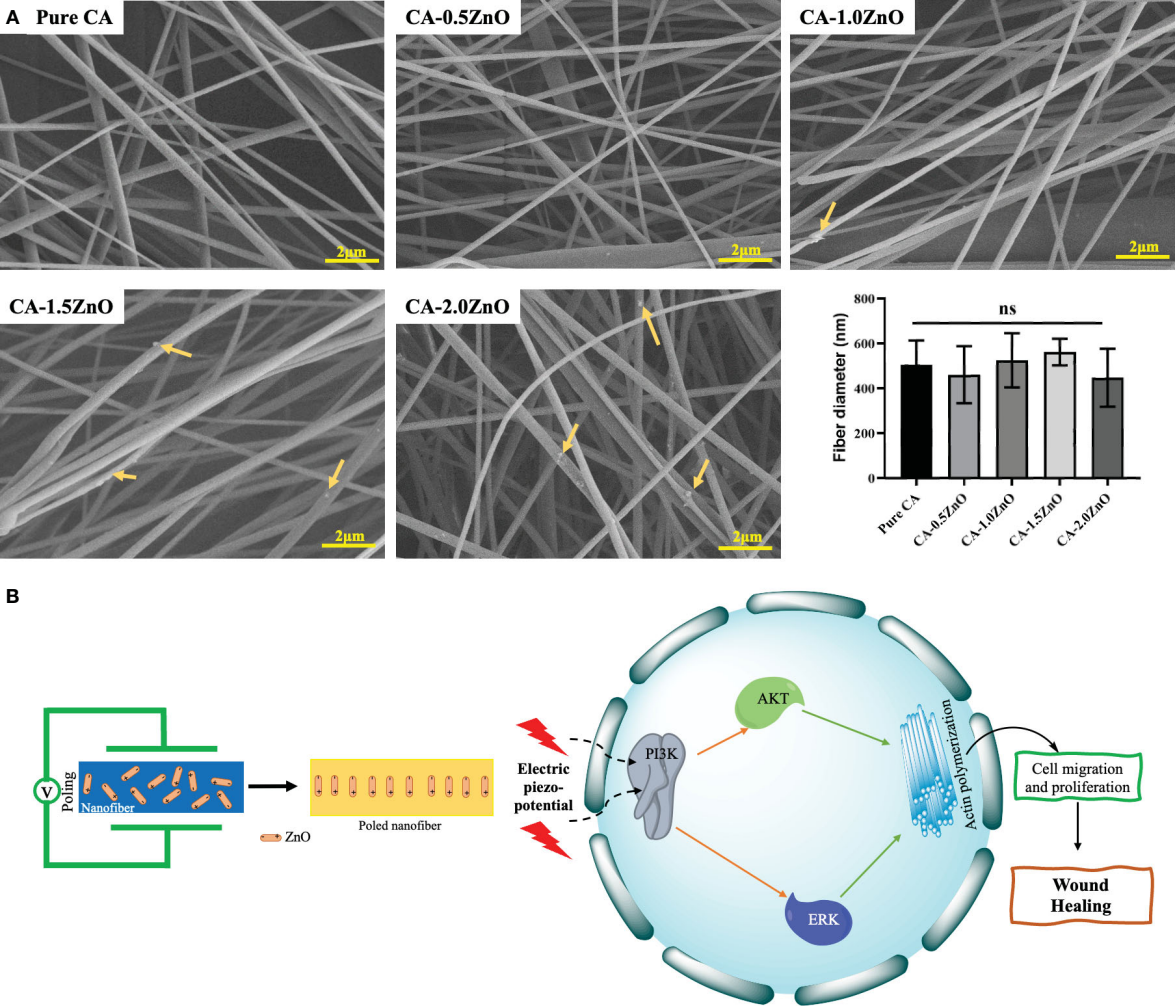
**(A)** FE-SEM images of the CA-ZnO nanofibers, **(B)** Schematics of potential wound healing mechanism of CA-ZnO piezoelectric nanofibrous mats. ns, non significant.

Recently, several researchers have extensively explored new classes of piezoelectric materials for their active involvement in the wound healing process, especially for the wound places in the dynamic mobile location (15, 16). It is evidenced that these piezoelectric materials generate electric potential (EP) under mechanical tensions. These EP further help to induce various steps of wound healing progression like proliferation and differentiation of fibroblasts, angiogenesis, collagen remodeling, etc. (**Figure 1B**) (17). For instance, a plethora of previous studies demonstrated that electric potential difference-derived electrical fields (EFs) generated from the piezo-potential to modulate skin cell behaviors (17–19). In the wound microenvironment, transepithelial possible variations (TEP) at the damaged epithelial layer generate an endogenous electrical field (EF). This EF is maintained until the skin regeneration process is complete. However, the differential ionic gradient at the wound site causes potential variations and disrupts TEP, influencing endogenous EF (19). By regulating skin cell activity and encouraging regeneration activities, this EF actively contributes to repairing skin wounds. Nonetheless, EP from the piezo-response also promotes several key steps during the wound healing process, such as angiogenesis, keratinocyte migration, myofibroblast differentiation, and fibroblast proliferation (17, 19).

Several metal oxides like TiO_2_, ZnO, Fe_2_O_3_, AgO_2_, and CuO_2_ are widely investigated in this regard (18, 20). ZnO is one of the most promising candidates for making the physiologically active scaffold due to its excellent piezoelectric coefficient and non-toxic and antibacterial activity (21). In the physiological environment, the Zn undergoes ionization and produces Zn^2+^; this ionic Zn stimulates collagen remodeling and cellular proliferation by inducing the voltage-gated Ca^2+^ ion channel through its piezoelectricity. For instance, Bhang et al. (19) fabricated a piezoelectric dermal patch composed of ZnO nanorods. They demonstrated that the patch can promote faster wound healing by enhancing cellular metabolism, cell migration, and protein synthesis. However, the patch consisted of PDMS and PEDOT: PSS, which are non-biodegradable and toxic to the host cells (22, 23).

Thus, the current study aims to investigate the effect of incorporating different concentrations of ZnO (0.5%, 1%, 1.5%, and 2%) into electrospun scaffolds composed of biodegradable CA and pristine CA. The resulting CA-ZnO electrospun fibrous mats were characterized using Field Emission Scanning Electron Microscopy (FESEM), Fourier-transform infrared spectroscopy (FTIR), and Thermogravimetric Analysis (TGA) to assess surface topology, chemical interactions, and its thermo-stability, respectively. Moreover, the porosity, hemolysis assay, *in vitro* cytotoxicity, and cell proliferation and migration characteristics of these composite mats were also assessed in this study.

## 2 Materials and methods

### 2.1 Chemicals

Zinc oxide (ZnO) (amorphous, particle size: <100 nm) was acquired from Sigma Aldrich, INDIA. The powder is used in its pristine state for every study without any further modifications. Another polymer, Cellulose acetate (CA) as flakes state (Acetic acid content: 53.5-56%), was acquired from Loba Chemie. If not explicitly mentioned, all the other chemicals are laboratory grade and used as obtained.

### 2.2 Electrospinning of CA-ZnO nanofibrous mats

ZnO-incorporated CA nanofibers were electrospinning by a traditional electrospinning setup (ESpin Nano, Chennai, India). Briefly, CA was first dissolved in acetone: N, N-Dimethylacetamide (2:1) at RT with vigorous stirring overnight. After that, ZnO was added in various amounts (0.5, 1, 1.5 & 2 wt %) and dispersed thoroughly by sonication. After that, the viscous polymeric solution was poured into the syringe, and the needles were connected to the electrode of the electrospinning machine. We have optimized various process parameters such as applied voltage, polymer concentration, flow rate, humidity, and temperature to obtain the continuous uniform fiber and bead-free mesh. The optimized parameters of the electrospinning process are described in **Table 1**. The fabricated 0.5% w/v ZnO reinforced Cellulose acetate (CA-0.5ZnO), 1% w/v ZnO reinforced Cellulose acetate (CA-1.0ZnO), 1.5% ZnO reinforced Cellulose acetate (CA-1.5ZnO), and 2% ZnO reinforced Cellulose acetate (CA-2.0ZnO) were used to denoted various CA-ZnO nanofibrous mats.

**Table 1:** Optimized electrospinning parameter for fabricating CA-ZnO nanofibrous mats.

Electrospinning parameters	
Solvent	Acetone : DMAc (2:1)
CA-ZnO concentration	15% (w/v)
Applied voltage	18 kV
Collector distance	20 cm
Flow rate	1 mL/h
Temperature	32°C
Humidity	45%

### 2.3 Characterizations of the prepared nanofibrous mats

#### 2.3.1 Fiber surface morphology

To examine the surface topology, fabricated CA-ZnO nanofibrous mesh was characterized by field emission-scanning electron microscopy (FE-SEM, Hitachi, Japan). Briefly, the nanofiber specimens were placed onto the glass slide, sputter coated with gold, and images were taken on multiple areas of the samples. The average diameter of the nanofibers was quantified using ImageJ software.

#### 2.3.2 Chemical interaction studies- ATR-FTIR

To examine the different chemical interactions and functional groups on the nanofibrous mats Attenuated Total Reflection-Fourier-transform infrared spectroscopy (ATR-FTIR) was employed (ALPHA Bruker, Germany). The samples were scanned from 4000 cm^-1^ to 600 cm^-1^ (24).

#### 2.3.3 Thermal characterization

The thermostability and melting points of the prepared nanofibrous mats were determined using thermogravimetric analysis (TGA, Netzsch, Germany). The nanofibrous samples (~2mg) were placed on the TGA plate and heated in the range of 25 to 500°C under dry nitrogen conditions (20) for reference empty plate was used. The data was acquired through Proteus 7.1 (Netzsch, Germany) software.

#### 2.3.4 Mechanical testing

The mechanical properties of the CA-ZnO nanofibrous mesh were investigated using a tensile testing machine (Tinios Olsen). Briefly, the membranes were cut in a dimension of 1×3 cm^2,^fixed in the adaptor, and stretched with a 5 mm/min speed using a 50 N load cell. The tensile strength, Young’s modulus, and % elongation were calculated from the stress vs strain curve.

#### 2.3.5 Water absorption test

The wettability of the fabricated nanofibers membrane was studied by immersing the samples (1 × 1 cm^2^) in PBS (pH 7.4) for 30 min. The weight of the specimens was taken before and after the test. The degree of swelling was calculated by.


$$\text{Degree of swelling}\left(\%\right)\;=\left(\left({\text{W}}_{\text{f}}-{\text{W}}_{\text{i}}\right)\right)/{\text{W}}_{\text{i}})\times 100$$

Where Wi & W_f_ are the initial and final weight of the sample, respectively.

#### 2.3.6 Porosity determination

The porosity of the nanofibrous mats was studied using the traditional solvent replacement method (25). The nanofibrous specimens (1×1 cm^2^) were weighed (W_i_) and soaked in 100% ethanol for one h. After that, the weight was retaken (W_f_), and the percentage of porosity was calculated by-


$$\text{Porosity}\left(\%\right)\;=\left(\left({\text{W}}_{\text{f}}-{\text{W}}_{\text{i}}\right)\right)/{\text{W}}_{\text{i}}\;\times 100$$

#### 2.3.7 Degradation study

The definitive degradation study in PBS determined the biodegradability of the fabricated scaffolds. Briefly, the nanofibrous specimens (1×1 cm^2^) were initially weighed (W_i_) and immersed in 10mL PBS (pH 7.4) for the 3, 5, and 7-days period. Then, after the specified time interval, the samples were taken from the PBS and weighed (W_f_) after drying in a vacuum oven. The study was done in triplicate, and the following equation calculated the percentage of swelling:


$$\text{Rate of Degradation}\left(\%\right)\;=\left(\left({\text{W}}_{\text{f}}-{\text{W}}_{\text{i}}\right)\right)/{\text{W}}_{\text{i}})\times 100$$

#### 2.3.8 Electrical characterizations – piezoelectric coefficient determination

The piezoelectric coefficient (d_33_) was determined using Berlincourt-type d_33_ (Piezotest-PM200, UK) meter (26). Briefly, the specimens (0.5 cm^2^) were subjected to the corona poling process within an 8 kV electric field for 30 min. After that, the poled sample was appropriately fixed between the piezometer gauzes. The coefficient value was determined from the front panel of the apparatus. For analysis purposes, five samples are analyzed from each group, and the average values are calculated.

### 2.4 Hemolysis assay

The blood compatibility of the electrospun membranes was tested by following the ASTM F-756 standard (26). The 500 μL blood was collected from the tail of BALB/c mice in a tube containing heparin solution as per the approval from IAEC/2020/034, NIPER Ahmedabad. After that, the RBC suspension was separated by centrifuging the tube at 2500 rpm for 15 minutes. Next, the RBC suspension was diluted 10 times with PBS and transferred to the sample tube. Samples were prepared by cutting the composite film into 1×1×0.5 cm^3^ dimensions. Subsequently, a mixture of 1.6 ml of deionized water and 400 μL RBC suspension was taken as a positive control, and another mixture, along with 400 μL RBC suspension, was taken as a negative control. All sample-containing tubes were then incubated for a further two hours at 37°C. Then the tubes were centrifuged at 2,500 rpm for 10 minutes, and the supernatant’s absorbance was measured at 541 nm using a UV-visible spectrophotometer. The % hemolysis was determined by-


$$\%\text{ hemolysis}=(\left({\text{A}}_{\text{s}}-{\text{A}}_{\text{p}}\right)/\left(\left({\text{A}}_{\text{s}}-{\text{A}}_{\text{n}}\right)\right)\times 100$$

Where, A_s_, A_p_ and A_p_ are denoted for the absorbance of the specimens, positive and negative control, respectively.

### 2.5 *In-vitro* cytotoxicity assessment

Biocompatibility of the developed CA and CA-ZnO electrospun scaffold has been evaluated according to ISO 10993-5 guidelines using Alamar blue assay (27). In this experiment, the red color Resazurin which is the principal active ingredient of Alamar blue is converted into the purple color Resorufin by the enzyme mitochondrial oxidoreductase. Before the study, the samples were sterilized by ethanol washing (10-90%) followed by 2h UV exposure. Later, the specimens were incubated for 3 days with the cell culture media, i.e., DMEM, and extracted solutions were collected for the experiments.

In brief, 20 μl of extract of different nanofibrous membranes containing various ZnO amounts (i.e., 0.5 – 2.5 wt %) was given to NIH 3T3 mouse embryo fibroblast cells (1×10^6^) and incubated for 1, 3, and 7 days at 37°C and 5% CO_2_. After the specific time interval, 0.5% w/v Alamar blue reagent was added to the media and further incubated for 4 h. At last, the resulting solution was analyzed by fluorescence spectroscopy at 560-590nm using a Multimode plate reader (Varioskan, Finland).

### 2.6 Cell adhesion assay

The cell adhesion characteristics of the electrospun membranes were studied using Scanning Electron Microscopy (SEM) (26). In brief, 1×10^6^ cells of NIH 3T3 were directly seeded on the top of the sterilized poled CA-ZnO membrane and incubated for 24 h at 37°C with a 5%CO_2_ environment. Then the samples were washed with PBS, and attached cells were fixed using an ice-cold 4% glutaraldehyde solution. After that, the specimens were sputtered gold coated and imaged using SEM. For calculating the percentage of cell adhesion area, images were taken from 3 random regions and analyzed using ImageJ.

### 2.7 *In-vitro* cell migration assessment

Scratch assay was used to investigate piezoelectric CA-ZnO nanofibrous mats’ cell migration and wound healing potential (28). For the experiment, sterile poled electrospun CA and ZnO-CA scaffold mats were placed into the serum-free DEME media, and after 24 h, extracts were collected. Then, 1× 10^5^ 3T3 fibroblast cells were seeded in six-well plates for 24 hours to form a monolayer. Next, a straight scratch was produced using 200 μL tips, and the unattached cells were subsequently removed by washing with PBS. Later, the prepared extract was added to the culture and incubated. The cell migration was observed at different time points, such as 0, 2, and 4 days and images were acquired by an optical microscope (Zeiss, Germany).

### 2.8 Statistical analysis

The experiments were conducted in triplicate, and the data were presented as the mean ± standard deviation. Statistical analysis was performed using GraphPad Prism software (Version 8.5, CA, USA) with a one-way ANOVA test. A significance level of P<0.05 was used to determine statistical significance. For the statistical analysis, ns, *, **, & *** denoted for non significant, P<0.05, P<0.01, P<0.001, respectively.

## 3 Results

### 3.1 Surface morphology

The optimized electrospinning parameters for obtaining a uniform, continuous, bead-free CA-ZnO were reported in **Table 1**. The SEM images of CA nanofibers with varying ZnO concentrations are depicted in **Figure 1A**. It has been found that all the prepared nanofibers membranes had an average diameter of 547- 608 nm. We can also confirm that varying concentrations of ZnO at low levels don’t affect the nanofiber diameter and surface topography.

Furthermore, incorporating ZnO in the nanocomposite is noteworthy as it leads to an enhancement in conductivity. Conductivity exhibits an inverse relationship with fiber diameter and behaves oppositely to viscosity. Specifically, increased viscosity results in a larger fiber diameter (24). These critical attributes influence the fiber diameter, ultimately resulting in a subtle change. However, this effect remains imperceptible due to the deficient concentration of ZnO.

### 3.2 Chemical interaction assessment-ATR-FTIR

**Figure 2:**
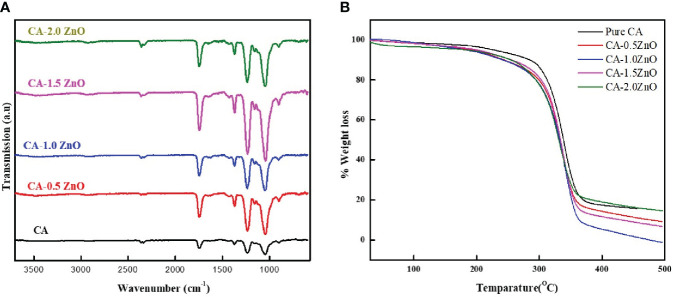
**(A)** ATR-FTIR spectrum and **(B)** TGA thermograph of the fabricated CA-ZnO nanofibrous electrospun mats.

Different chemical and functional groups’ interactions between CA and ZnO were investigated by ATR-FTIR spectroscopy. As illustrated in **Figure 2A**, the pure CA membrane consists of all the characteristics bands of Cellulose acetate, such as 1742 cm^-1^ and 1043 cm^-1,^ attributed to the stretching vibration of C=O of ester groups and asymmetric stretching of C-O-C from the sugar moieties of CA (29). After incorporating ZnO, all the peaks are shifted slightly because of the electrostatic interaction of negatively charged surfaces of ZnO with several -OH groups present on the CA. Moreover, from the FTIR spectra, it has been concluded that in the nanofibrous membrane, the semicrystalline nature of ZnO is adequately maintained, which is essential for its piezoelectric property (30, 31). Furthermore, no definite chemical interaction was found between CA and ZnO, as all the typical characteristic peaks of CA were present in the resulting nanofibrous mats.

### 3.3 Thermal characterization

TGA examined the thermal stability of the fabricated CA-ZnO nanofibrous electrospun mats. From the TGA thermogram depicted in **Figure 2B**, 5% decomposition of the pure CA fibers started from 183.1°C whereas, after the reinforcement of the 0.5, 1, 1.5, and 2% ZnO, it was found to be 172.4, 164.8, 167.2 and 153.5°C, respectively. Thus, from the TGA analysis, it was confirmed that the ZnO incorporation into the pristine does not result in any thermal interaction and does not significantly affect the thermal stability of the scaffolds.

### 3.4 Mechanical properties

**Figure 3:**
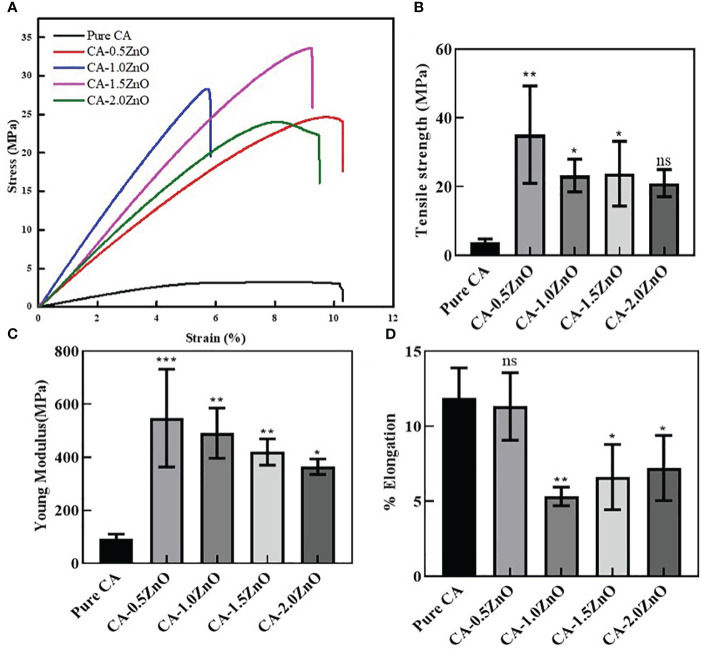
Mechanical characterizations of the fabricated CA-ZnO nanofibrous electrospun mats. **(A)** stress vs. strain curve, **(B)** Tensile strength, **(C)** young modulus, and **(D)**% elongation comparison of CA-ZnO membranes with pure CA. ns, non significant, *P<0.05, **P<0.01, ***P<0.001.

The ideal wound healing substrates require sufficient mechanical integrity to support the damaged body tissue to heal and provide a platform for cell and ECM formation, protection from external injury, etc. Therefore, several mechanical characteristics of the developed nanofibrous membranes were examined from the stress vs. strain curve, depicted in **Figure 3**. As shown, after the reinforcement of ZnO into the CA, the tensile strength and Young’s modulus of the nanofibrous scaffolds have increased with the increased concentrations of ZnO. In contrast, the %elongation has decreased significantly after the addition of 1% ZnO into the CA. Notably, the young modulus of the different ZnO-reinforced nanofibers decreased when we increased the amount of ZnO. Moreover, the ZnO enforcement into the CA fibers significantly enhanced the mechanical characteristics, assisting the healing process.

### 3.5 Water absorption test

**Figure 4:**
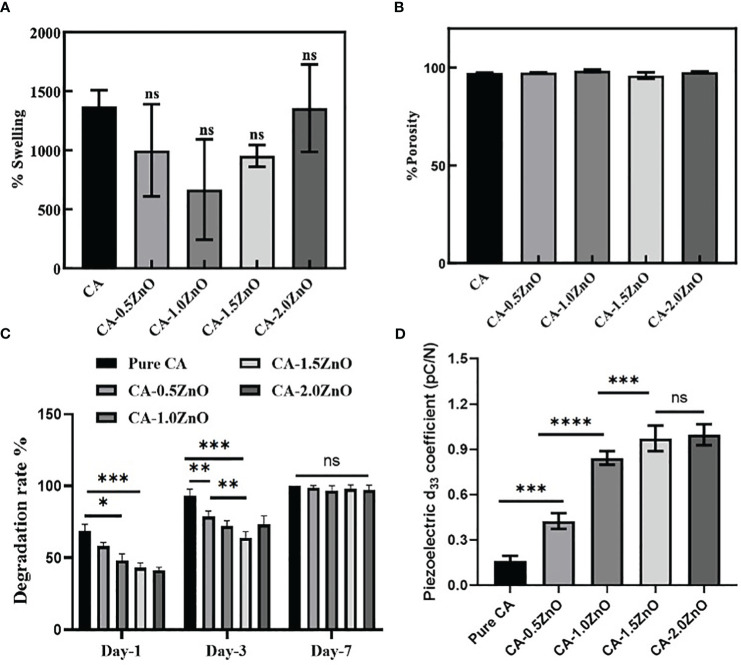
**(A)** Water absorption capacity of the fabricated nanofibrous mats, **(B)** porosity of the electrospun membranes, **(C)** rate of degradation of the CA-ZnO electrospun mats, and **(D)** Piezoelectric d_33_ coefficient of the CA-ZnO nanofibrous electrospun mats. ns, non significant, *P<0.05, **P<0.01, ***P<0.001, ****P<0.001.

Evaluation of water uptake capacity is crucial for designing wound dressings and other biomedical applications. To be an ideal wound dressing material, it should have the properties to absorb the dead cellular debris and wound extrudates (32). The previous studies noted that the recommended range for a wound dressing’s fluid absorption capability is 100 to 900% (33). Thus, the water uptake capacity of the developed electrospun specimens was evaluated and depicted in **Figure 4A**. As shown, it has been demonstrated that all the samples exhibited more than ~600% water uptake capacity. There is no change in the swell ability of the CA-ZnO electrospun mats compared to pristine CA fibers.

### 3.6 Porosity determination

The porous microstructure of the nanofibrous mats is one of the crucial prerequisites for cellular crosstalk as the porous scaffolds exhibit high surface area and are an excellent medium for nutrient exchange. **Figure 4B** represents the permeable characteristics observed in all fabricated electrospun scaffolds. However, it can be observed that the porosity of the platforms with the inclusion of ZnO did not exhibit a significant difference compared to the pure CA-nanofibrous mats. Furthermore, all the electrospun mats displayed a high level of porosity, ranging from 98% to 100%, which is highly beneficial to the cell’s adhesion and proliferation.

### 3.7 Degradation study

Bio-degradation capacity of the wound closure devices offers a conducive microenvironment for tissue healing by providing nutrients from the degraded by-products and giving space for the recruitment of new cells at the wound site. The biodegradation capability of the fabricated CA-ZnO electrospun mats is stated in **Figure 4C**. As illustrated, all the samples were fully degraded within 7 days. However, on day 1, it was found that compared to the pure CA, which contaminated over 63.24%, the ZnO-reinforced electrospun mats degraded 52.25%, 47.73%, 39.2%, and 37.67%, respectively. On a similar trend, on day 3, it was observed that ZnO-incorporated specimens exhibited a significantly lower degradation rate compared to pure CA samples. Furthermore, it has also been witnessed that the degradation rate is decreased with the increase of ZnO concentration. However, the maximum ZnO concentrated samples, i.e., CA-2.0ZnO, exhibit an increase in the degradation rate, which may be due to the aggregation of ZnO clusters preventing the proper association of ZnO molecules with CA-fibers. At last, on the 7th day, we can observe the complete degradation of all the specimens. Nonetheless, previous reports also indicated that the degradation process of the CA is predominantly governed by hydrolysis and enzymatic degradation in the physiological environment (12). Moreover, the suitable timeline of the degradation pattern of the fabricated nanofibrous membranes gives a favorable condition for accelerating the healing process.

### 3.8 Piezoelectric coefficient determination

In principle, the d_33_ coefficient of piezoelectric material directly represents the amount of electric potential generated onto the material surface when subjected to the unit load in the same direction (19). Therefore, the piezoelectric characteristics of the fabricated electrospun mats were evaluated by measuring the d_33_ coefficient and reported in **Figure 4D**. It has been found that compared to pure CA, all ZnO-reinforced fibers exhibit a higher d_33_ coefficient. The pure CA nanofibers also possess a natural d_33_ coefficient of 0.21-0.27 pC/N, whereas, after the ZnO reinforcement, the d_33_ coefficient increased significantly to 0.52 ± 0.04, 0.87 ± 0.03, 0.94 ± 0.02, and 0.92 ± 0.02 pC/N for CA-0.5ZnO, CA-1.0ZnO, CA-1.5ZnO, and CA-2.0ZnO, respectively.

### 3.9 Hemolysis assay

**Figure 5:**
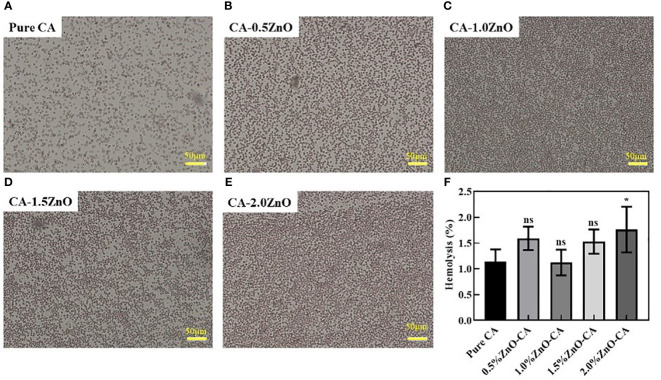
Hemolysis activity of the CA-ZnO nanofibrous mats. **(A–E)** Microscopic images of the RBCs after contact with CA-ZnO nanofibrous mats. **(F)** quantitative analysis of the hemolysis activity. ns, non significant, *P<0.05.

Blood compatibility is one of the most fundamental features of any wound healing scaffold, as it gives first-hand information regarding blood coagulation in the wound site and biocompatibility. Thus we have examined the blood compatibility of the fabricated nanofibrous mats by incubating the fibers with RBC, as shown in **Figure 5**. As observed, Pure CA, CA-0.5ZnO, CA-1.0ZnO, and CA-1.5ZnO scaffolds exhibited %hemolysis below 2%, whereas the CA-2.0ZnO nanofibers have significantly higher hemolysis capability.

### 3.10 Cytotoxicity assessment

**Figure 6:**
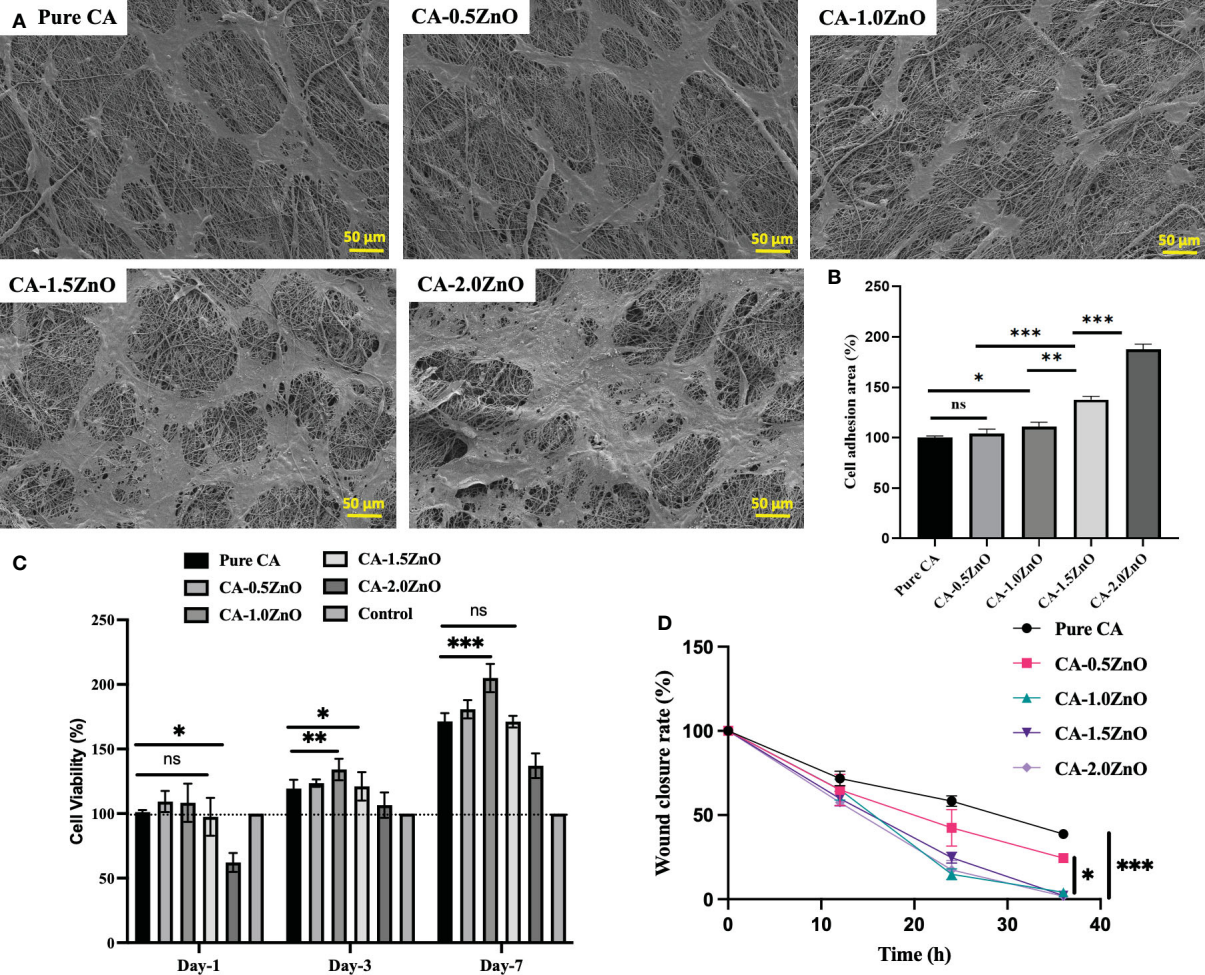
**(A)** SEM images of NIH 3T3 mouse embryo fibroblasts cells adhesion over the CA-ZnO electrospun mats after 24h incubation, **(B)** Percentages of Cell adhesion area, **(C)** quantitative % cell viability of NIH 3T3 cells over the CA-ZnO electrospun mats on 1, 3 and 7th day. **(D)** Wound closure rate ater CA-ZnO electrospun scaffolds application. ns, non significant, *P<0.05, **P<0.01, ***P<0.001.

The biocompatibility and cell proliferation characteristics of the prepared nanofibrous sheets were determined by Alamar blue assay, and the % cell viability is reported in **Figure 6C**. Nevertheless, in the current study, we have specifically used NIH 3T3 fibroblasts cells for the investigation, as fibroblasts are one of the most abundant and first lines of cells affected during skin wound healing. As shown, on day-1, all the mats are shown no significant difference in cell viability except CA-2.0ZnO mats. This may be due to the potential cytotoxic effect of ZnO through its higher-charged surface, which damages the cell wall (21). However, on Day-3&7, all the fibers exhibited higher cell viability than the pristine CA. It has also been observed that at the end of the 7th day, CA-1.0ZnO showed the highest %cell viability of all the other fibers. In addition, after the 7 days, all the fabricated nanofibrous membranes exhibit over ~150% cell viability compared to day-1. These results indicated the cell proliferation capability of the fabricated scaffolds.

### 3.11 Cell attachment

Adhesion of the cells onto the scaffold surface facilitated the healing process by inducing cell-cell interaction, more ECM production, etc., eventually leading to the regeneration of new tissues. **Figure 6A** depicted the SEM images of fibroblast cell adhesion over the fabricated piezoelectric fibers respectively. Cell attachment is quite prominent, as represented in the electrospun mats. **Figure 6B** illustrates each specimen’s percentage cell adhesion area. As indicated, 1%, 1.5%, and 2% ZnO-reinforced CA mats exhibit at least ~30%, 52%, and 100% enhancement in cell adhesion area compared to pure CA mats. The enhanced cell adhesion on the poled piezoelectric nanofibers is due to the surface piezo-potential of the nanofibers, which interacts with the voltage-gated ion channels present on the cell surface and upregulate the collagen synthesis process and F-actin formation, which are crucial for the cell adhesion.

### 3.12 Wound healing scratch/cell migration assessment

**Figure 7:**
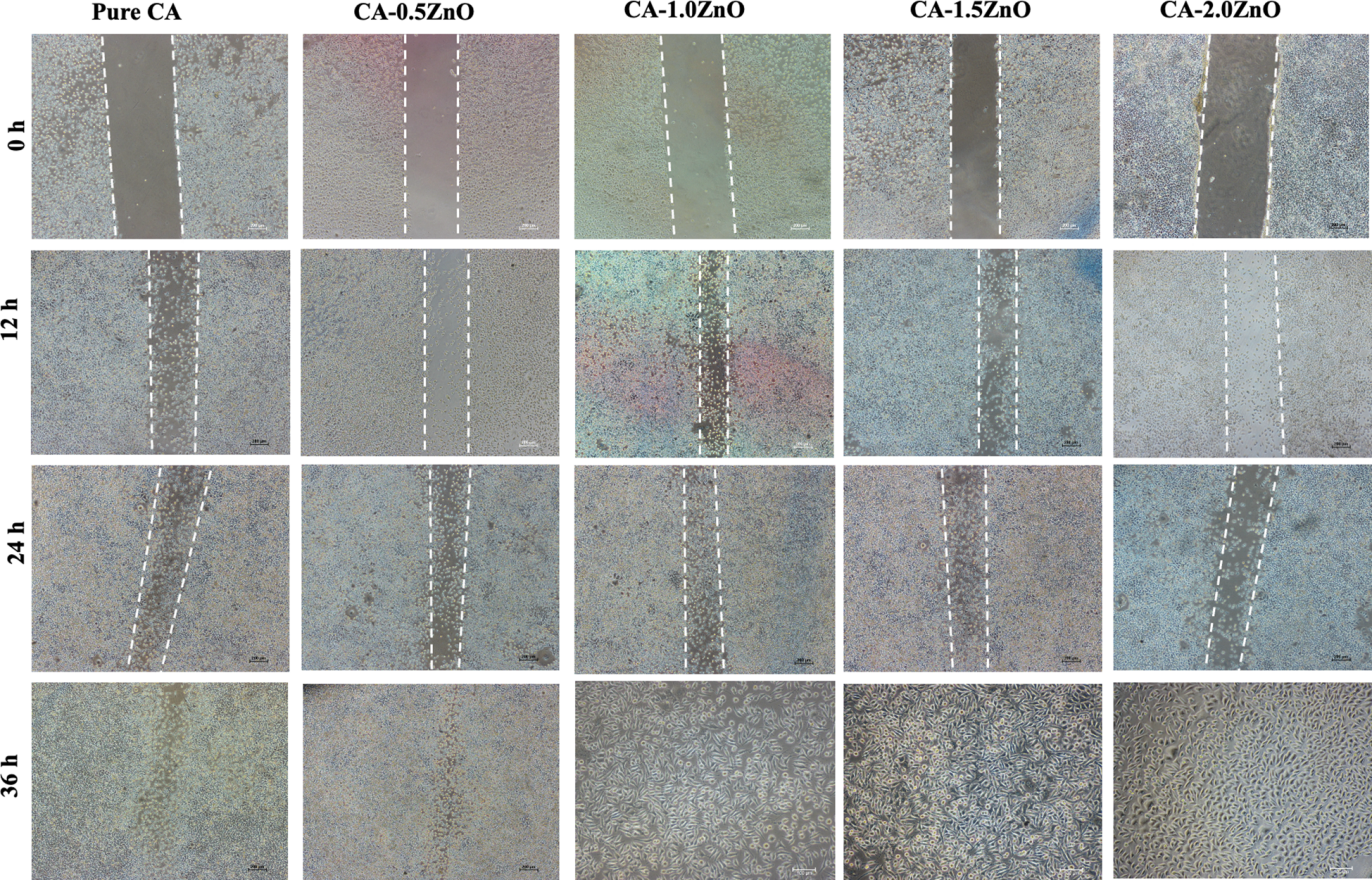
Microscopy images of Cell migration scratch assay.

Cell migration assay with fibroblast cells evaluated the piezoelectric mats’ potential to heal wounds. As depicted in **Figure 7**, compared to the pristine CA mats, CA0.5ZnO does not exhibit a significantly faster rate. In contrast, all the other mats, i.e., CA-1.0ZnO, CA-1.5ZnO, and CA-2.0ZnO, demonstrated substantially faster wound healing within 36 h period (**Figure 6D**).

This is because of the piezoelectric potentials generated from the poled nanofibrous mats sensed by the adjacent cells, and the polarization helps cells to migrate faster by inducing the PI3K-AKT pathways (19). Nevertheless, a previous study by Bhang et al. (19) demonstrated that upon EF stimulation generated from the piezo-potential, PI3K, one of the first molecules that drive cell electrotaxis, which enabling cells to produce a membrane protrusion from the leading edge (19). Consequently, PI3K phosphorylation increases Akt phosphorylation and causes Akt distribution in the direction of the cells’ migration. Moreover, the poled piezo-electric nanofiber modulates the cellular crosstalk and promotes cell migration for faster healing.

## 4 Discussion

Being a complex, dynamic physiological process, wound healing is among the most common healthcare burdens (34). Previously, the porous electrospun-based scaffolds have been shown to enhance cellular infiltration and adhesion by generating a nano/micro topological microenvironment for cell-biomaterial interaction, enhancing cellular infiltration and attachment (35, 36). The interfibrous space in the nanofibrous membranes appears to influence cell communication and improve interactions with the extracellular microenvironment (37). However, most currently reported electrospun fibers exhibited their biological functionalities based on their ECM mimicry, nano-architecture, porous degradable characteristics, and lack of active participation in the natural healing (38).

In the current study, we have prepared the piezoelectric electrospun fibers by reinforcing traditional piezoelectric material, i.e., ZnO, and optimized its piezoelectrical performance for efficient wound healing. Besides being an FDA-approved naturally derived polymer, CA has been widely explored in the clinical setup as a traditional dressing material for wound cleaning and gauging. In this current study, we have investigated the smart, mechano-responsive, piezoelectric characteristics of the CA nanofibrous membrane in combination with ZnO. From the nanofibrous scaffolds’ SEM images, it is evident that the fabricated fibers mimic the structural organization of the natural extracellular matrix (ECM) present in the skin (39). The electrospun nanofibers have substantial porosity and a conducive structural environment (19). Nevertheless, the scaffold’s porous nature, as seen in SEM pictures, would facilitate the interchange of vital nutrients and cellular waste throughout the healing process (40, 41).

From the collective evidence of FTIR-ATR and DSC analysis, it has been confirmed that the ZnO is successfully reinforced into the pristine CA-fibers during the electrospinning process, and the semi-crystalline structure of the ZnO is also thermally stable and preserved in the final electrospun mats, which is essential for eliciting the piezoelectric performance (19) (**Figure 1B**).

Mechanical properties are one of the most crucial parameters for any wound closure appliance to protect it from external damage and provide structural support in tissue healing (42). Therefore, we have characterized the fabricated nanofibrous mats in terms of their tensile strength, Young’s Modulus, and % elongations. Mechanical characterizations of the various ZnO-reinforced CA-fibers revels the concentration-dependent modulation of tensile strength, Young Modulus, and % elongation. It has been found that up to ~1.5% ZnO reinforcement enhances the tensile strength of the mats, but 2% reinforcement significantly reduces the same compared to pure CA mats. Although a significant increment of Young Modulus has been observed after the ZnO addition, it has been found that as the concentration of ZnO increases, the modulus has decreased with respective ZnO percentages. This may be due to the aggregation of the ZnO, which affected the stress propagation over the nanofibrous mats and resulted in the decrement modulus (42). This phenomenon has also been observed in the % elongation characteristics of the mats. The increment in the amount of semicrystalline ZnO resists the polymer chain elongation, decreasing the extension. The enhanced tensile strength of the ZnO-reinforced specimens is attributed to the H-bond interactions between the CA and ZnO surface charges.

From the water swell ability index, it has been evident that the ZnO incorporation doesn’t affect the nanofibrous mats’ water absorption and retention ability. This is important to note that during the wound healing process, damaged tissue/cells secrete several kinds of inflammatory cytokines with the body fluids (43). Absorption of this necrotic tissue debris passively clears the wound site and helps to recruit the other cells, such as macrophages and fibroblasts, for faster wound healing (44). Nevertheless, the moisture retention ability of the nanofibrous mats is also helpful for maintaining moisturization in the wound site for better cell survival (45). The porous networks of the nanofibrous membrane augment the extracellular matrix of the natural body tissue, which substantially helps for better cell adhesion and consecutive collagen synthesis needed for tissue healing (39).

Besides being swellable, biodegradation characteristics are another crucial feature of nanofibrous scaffolds. The degradation study demonstrated that all the nanofibrous specimens were wholly degraded within seven days. However, for the comparison, it has been noted that on days 1 and 3, the 0.5-1.5% ZnO reinforced samples have shown significantly slower degradation; on the contrary, 2% ZnO-CA samples exhibit higher degradation behavior which is similar to pure CA. This is due to the phase separation of the aggerated ZnO particles from the CA fibers. Notably, previous studies demonstrated that the CA is pre-dominantly degraded by the protease enzymes and results in non-toxic by-products. On the other hand, ZnO was utilized as a nutrition source for the zinc, and after that, the excessive amount was cleared through the rental route. Moreover, both of the components of the nanofibrous mats are completely suitable for the wound healing process.

The d_33_ piezoelectric coefficient measurement is evident in the piezoelectric properties of the fabricated poled nanofibers (46). In this current work, we have previously chosen cellulose as a parent fibrous mat due to its already confirmed piezoelectric properties. Song et al. (47) reported that natural cellulose could also exhibit piezoelectric properties due to its crystal structure and a d_33_ of around 0.2-0.8 pC/N, similar to natural collagen. The enhanced piezoelectric performance after the ZnO reinforcement into the fiber matrix is due to the crystalline structure of the ZnO (48). Moreover, the fabricated poled nanofibers exhibited higher piezoelectric performance, which can activate the critical downstream pathways of piezoelectric-based PI3K-AKT pathway for the collagen production and fibroblasts migration for the faster wound healing and regeneration process (**Figure 1B**) (19).

Furthermore, we have also investigated the cell attachment and cell migration ability of the poled piezoelectric fibers *in-vitro*. The piezo-electric potential generated on the surface of the poled nanofibers after mechanical stimulation drives the wound healing process through enhanced cellular metabolism, migration, and protein synthesis. However, there still are some limitations in this study as the therapeutic effects of the piezoelectric nanofibers can vary depending on the age, wound type, wound site, and wound size of the patients. Taken together, as discussed above, the enhanced piezoelectric potentials of the poled piezoelectric ZnO-CA nanofibrous mats interact with the cells and modulate the wound microenvironment to fasten the healing process (49); on top of that, the well-established antimicrobial role of the ZnO also contributes additional advantages for preventing the wound site from microbial infections (50, 51).

## 5 Conclusion

The electrospinning techniques successfully prepared piezoelectric-based smart ZnO-CA nanofibrous mats. SEM images of the mats demonstrated the porous microstructure of the mats, which successfully augmented the extracellular matrix of the natural tissue. DSC and FTIR studies also show the thermal stability and chemical interactions between the reinforced ZnO and cellulose acetate. The enhanced mechanical properties of the ZnO-reinforced CA nanofibrous mats were confirmed by examining their tensile strength, Young’s modulus, and % elongation. Furthermore, the hemolysis study confirmed the hemocompatibility of the fabricated nanofibrous membrane. The enhanced piezoelectric potential proved the nanofibers’ piezoelectric performance due to cellulose’s natural piezoelectric properties and the crystal structure of ZnO.

At last, we have also found that the CA-1.0ZnO nanofibrous membranes have a clear degradation pattern needed for the wound healing timeline. Nonetheless, the SEM images of the cell adhesion and scratch assay demonstrated the efficacy of piezoelectricity during the faster wound healing process. However, to get a more mechanistic insight into the piezoelectric potential to induce the physiological healing process, detailed *in-vivo* studies are required in future studies. Moreover, the current study revealed that the incorporation of ZnO into the cellulose acetate nanofibers exhibits a superior mechanical, porous, piezoelectrical, thermally stable, biocompatible, nanofibrous which can modulate the wound microenvironment, enhances the cell adhesion and faster cells migration which are essential for the wound healing.

## Data availability statement

The original contributions presented in the study are included in the article/supplementary material. Further inquiries can be directed to the corresponding author.

## Ethics statement

The studies involving animals were reviewed and approved by Institute animal ethics committee, National Institute of Pharmaceutical Education and Research-Ahmedabad, file no. IAEC/2020/034.

## Author contributions

Experimental, validation, formal analysis, and writing original draft preparation by SG, SV, and NM. Investigation, resources, writing review and editing, supervision, project administration by RV. Conceptualization, investigation, resources, writing review and editing, supervision, project administration, and funding acquisition by GK. All authors contributed to the article and approved the submitted version.

## References

[1] Khullar L Harjai K Chhibber S. Therapeutic and pro-healing potential of advanced wound dressings loaded with bioactive agents. Future Microbiol (2023) 18(1):43–63. doi: 10.2217/fmb-2022-0162 36537228

[2] Firoozbahr M Kingshott P Palombo EA Zaferanloo B. Recent advances in using natural antibacterial additives in bioactive wound dressings. Pharmaceutics (2023) 15(2):644. doi: 10.3390/pharmaceutics15020644 36839966 PMC10004169

[3] Yusuf Aliyu A Adeleke OA. Nano-fibrous scaffolds for diabetic wound healing. Pharmaceutics (2023) 15(3):986. doi: 10.3390/pharmaceutics15030986 36986847 PMC10051742

[4] Karthick SA Manjari K Devi MG. Biocompatible and bioactive PVA/Sericin/Chitosan nano-fibrous wound dressing matrix. Appl Surf Sci Adv (2023) 13:100362. doi: 10.1016/j.apsadv.2022.100362

[5] Ding Y-W Wang Z-Y Ren Z-W Zhang X-W Wei D-X. Advances in modified hyaluronic acid-based hydrogels for skin wound healing. Biomater Sci (2022) 10:3393–409. doi: 10.1039/D2BM00397J 35575243

[6] Yang Y Liang Y Chen J Duan X Guo B. Mussel-inspired adhesive antioxidant antibacterial hemostatic composite hydrogel wound dressing via photo-polymerization for infected skin wound healing. Bioact Mater (2022) 8:341–54. doi: 10.1016/j.bioactmat.2021.06.014 PMC8427086 34541405

[7] Sorg H Sorg CG. Skin wound healing: of players, patterns, and processes. Eur Surg Res (2022) 64(1):1–17. doi: 10.1159/000528271 36417847

[8] Janmohammadi M Nazemi Z Salehi AOM Seyfoori A John JV Nourbakhsh MS. Cellulose-based composite scaffolds for bone tissue engineering and localized drug delivery. Bioact Mater (2023) 20:137–63. doi: 10.1016/j.bioactmat.2022.05.018 PMC9142858 35663339

[9] Han X Chen L Yanilmaz M Lu X Yang K Hu K. From nature, requite to nature: Bio-based cellulose and its derivatives for construction of green zinc batteries. Chem Eng J (2023) 454:140311. doi: 10.1016/j.cej.2022.140311

[10] Peranidze K Safronova TV Kildeeva NR. Electrospun nanomaterials based on cellulose and its derivatives for cell cultures: recent developments and challenges. Polymers (2023) 15(5):1174. doi: 10.3390/polym15051174 36904415 PMC10007370

[11] Abu-Zurayk R Alnairat N Khalaf A Ibrahim AA Halaweh G. Cellulose acetate membranes: Fouling types and antifouling strategies—A brief review. Processes (2023) 11(2):489. doi: 10.3390/pr11020489

[12] Aziz T Farid A Haq F Kiran M Ullah A Zhang K. A review on the modification of cellulose and its applications. Polymers (2022) 14(15):3206. doi: 10.3390/polym14153206 35956720 PMC9371096

[13] Oberlintner A Huš M Likozar B Novak U. Multiscale study of functional acetylation of cellulose nanomaterials by design: ab initio mechanisms and chemical reaction microkinetics. ACS Sustain Chem Eng (2022) 10(47):15480–9. doi: 10.1021/acssuschemeng.2c04686

[14] Kumar M Ge Y Hilles AR Bhatia A Mahmood S. A review on polysaccharides mediated electrospun nanofibers for diabetic wound healing: Their current status with regulatory perspective. Int J Biol Macromol (2023) 234:123696. doi: 10.1016/j.ijbiomac.2023.123696 36801273

[15] Ji J Yang C Shan Y Sun M Cui X Xu L. Research trends of piezoelectric nanomaterials in biomedical engineering. Adv NanoBiomed Res (2023) 3(1):2200088. doi: 10.1002/anbr.202200088

[16] Weng Z Xu Y Gao J Wang X. Research progress of stimuli-responsive ZnO-based nanomaterials in biomedical applications. Biomater Sci (2023) 11:76–95. doi: 10.1039/D2BM01460B 36385188

[17] Ghosh S Qiao W Yang Z Orrego S Neelakantan P. Engineering dental tissues using biomaterials with piezoelectric effect: current progress and future perspectives. J Funct Biomater (2022) 14(1):8. doi: 10.3390/jfb14010008 36662055 PMC9867283

[18] Nikolova MP Chavali MS. Metal oxide nanoparticles as biomedical materials. Biomimetics (2020) 5(2):27. doi: 10.3390/biomimetics5020027 32521669 PMC7345077

[19] Bhang SH Jang WS Han J Yoon JK La WG Lee E. Zinc oxide nanorod-based piezoelectric dermal patch for wound healing. Adv Funct Mater (2017) 27(1):1603497. doi: 10.1002/adfm.201603497

[20] More N Ranglani D Hiray AR Kapusetti G. Piezoelectric ceramics as stimulatory modulators for regenerative medicine. Adv Ceramics Versatile Interdiscip Appl (2022) p:313–38. doi: 10.1016/B978-0-323-89952-9.00005-1

[21] Jaberifard F Ramezani S Ghorbani M Arsalani N Moghadam FM. Investigation of wound healing efficiency of multifunctional eudragit/soy protein isolate electrospun nanofiber incorporated with ZnO loaded halloysite nanotubes and allantoin. Int J Pharm (2023) 630:122434. doi: 10.1016/j.ijpharm.2022.122434 36435502

[22] Jia B Li G Cao E Luo J Zhao X Huang H. Recent progress of antibacterial hydrogels in wound dressings. Mater Today Bio (2023) 19:100582. doi: 10.1016/j.mtbio.2023.100582 PMC9988584 36896416

[23] Yaacob A Jamaludin NS. Biodegradable polymers for cardiac tissue engineering. In: Handbook of Biodegradable Materials. Springer, Cham (2023). p. 979–1013. doi: 10.1007/978-3-031-09710-2_44

[24] Siddheswaran R Sankar R Ramesh Babu M Rathnakumari M Jayavel R Murugakoothan P. Preparation and characterization of ZnO nanofibers by electrospinning. Crystal Res Technol: J Exp Ind Crystallography. (2006) 41(5):446–9. doi: 10.1002/crat.200510603

[25] Kandhasamy S Perumal S Madhan B Umamaheswari N Banday JA Perumal PT. Synthesis and fabrication of collagen-coated ostholamide electrospun nanofiber scaffold for wound healing. ACS Appl Mater Interfaces (2017) 9(10):8556–68. doi: 10.1021/acsami.6b16488 28221758

[26] Jacob J More N Mounika C Gondaliya P Kalia K Kapusetti G. Smart piezoelectric nanohybrid of poly (3-hydroxybutyrate-co-3-hydroxyvalerate) and barium titanate for stimulated cartilage regeneration. ACS Appl Bio Mater (2019) 2(11):4922–31. doi: 10.1021/acsabm.9b00667 35021492

[27] Wu B Wei C Hsueh N Ding SJ. Comparative cell attachment, cytotoxicity and antibacterial activity of radiopaque dicalcium silicate cement and white-coloured mineral trioxide aggregate. Int endodontic J (2015) 48(3):268–76. doi: 10.1111/iej.12310 24802368

[28] Liang C-C Park AY Guan J-L. *In vitro* scratch assay: a convenient and inexpensive method for analysis of cell migration *in vitro* . Nat Protoc (2007) 2(2):329–33. doi: 10.1038/nprot.2007.30 17406593

[29] Santos-Sauceda I Castillo-Ortega M del Castillo-Castro T Armenta-Villegas L Ramírez-Bon R. Electrospun cellulose acetate fibers for the photodecolorization of methylene blue solutions under natural sunlight. Polym Bull (2021) 78:4419–38. doi: 10.1007/s00289-020-03324-y

[30] Le AT Ahmadipour M Pung S-Y. A review on ZnO-based piezoelectric nanogenerators: Synthesis, characterization techniques, performance enhancement and applications. J Alloys Compounds (2020) 844:156172. doi: 10.1016/j.jallcom.2020.156172

[31] Yang X Daoud WA. Triboelectric and piezoelectric effects in a combined tribopiezoelectric nanogenerator based on an interfacial ZnO nanostructure. Adv Funct Mater (2016) 26(45):8194–201. doi: 10.1002/adfm.201602529

[32] Dhivya S Padma VV Santhini E. Wound dressings–a review. BioMedicine (2015) 5(4):22. doi: 10.7603/s40681-015-0022-9 26615539 PMC4662938

[33] Rezvani Ghomi E Khalili S Nouri Khorasani S Esmaeely Neisiany R Ramakrishna S. Wound dressings: Current advances and future directions. J Appl Polym Sci (2019) 136(27):47738. doi: 10.1002/app.47738

[34] DesJardins-Park HE Gurtner GC Wan DC Longaker MT. From chronic wounds to scarring: The growing health care burden of under-and over-healing wounds. Adv Wound Care (2022) 11(9):496–510. doi: 10.1089/wound.2021.0039 PMC9634983 34521257

[35] Sundaramurthi D Krishnan UM Sethuraman S. Electrospun nanofibers as scaffolds for skin tissue engineering. Polym Rev (2014) 54(2):348–76. doi: 10.1080/15583724.2014.881374

[36] Zhu X Cui W Li X Jin Y. Electrospun fibrous mats with high porosity as potential scaffolds for skin tissue engineering. Biomacromolecules (2008) 9(7):1795–801. doi: 10.1021/bm800476u 18578495

[37] Rahmati M Mills DK Urbanska AM Saeb MR Venugopal JR Ramakrishna S. Electrospinning for tissue engineering applications. Prog Mater Sci (2021) 117:100721. doi: 10.1016/j.pmatsci.2020.100721

[38] Agarwal S Wendorff JH Greiner A. Progress in the field of electrospinning for tissue engineering applications. Adv Mater (2009) 21(32-33):3343–51. doi: 10.1002/adma.200803092 20882501

[39] Ghomi ER Lakshminarayanan R Chellappan V Verma NK Chinnappan A Neisiany RE. Electrospun aligned PCL/gelatin scaffolds mimicking the skin ECM for effective antimicrobial wound dressings. Adv Fiber Mater (2023) 5(1):235–51. doi: 10.1007/s42765-022-00216-w

[40] Loh QL Choong C. Three-dimensional scaffolds for tissue engineering applications: role of porosity and pore size. (2013) 19(6):485–502. doi: 10.1089/ten.teb.2012.0437 PMC3826579 23672709

[41] Lutzweiler G Ndreu Halili A Engin Vrana N. The overview of porous, bioactive scaffolds as instructive biomaterials for tissue regeneration and their clinical translation. Pharmaceutics (2020) 12(7):602. doi: 10.3390/pharmaceutics12070602 32610440 PMC7407612

[42] Dai H Sun T Han T Guo Z Wang X Chen Y. Aggregation behavior of zinc oxide nanoparticles and their biotoxicity to Daphnia magna: Influence of humic acid and sodium alginate. Environ Res (2020) 191:110086. doi: 10.1016/j.envres.2020.110086 32846168

[43] Schultz GS Chin GA Moldawer L Diegelmann RF. 23 principles of wound healing. Mechanisms of vascular disease: a reference book for vascular specialists. Adelaide (AU): University of Adelaide Press (2011) 423. doi: 10.1017/UPO9781922064004.024 30485016

[44] Junker JP Kamel RA Caterson E Eriksson E. Clinical impact upon wound healing and inflammation in moist, wet, and dry environments. Adv Wound Care (2013) 2(7):348–56. doi: 10.1089/wound.2012.0412 PMC3842869 24587972

[45] Yamashita Y Ohzuno Y Saito Y Fujiwara Y Yoshida M Takei T. AutoclavingTriggered hydrogelation of chitosan-gluconic acid conjugate aqueous solution for wound healing. Gels (2023) 9(4):280. doi: 10.3390/gels9040280 37102892 PMC10137746

[46] Guo Q Cao G Shen I. Measurements of piezoelectric coefficient d33 of lead zirconate titanate thin films using a mini force hammer. J Vib Acoust (2013) 135(1). doi: 10.1115/1.4006881

[47] Song Y Shi Z Hu G-H Xiong C Isogai A Yang Q. Recent advances in cellulose-based piezoelectric and triboelectric nanogenerators for energy harvesting: a review. J Mater Chem A (2021) 9(4):1910–37. doi: 10.1039/D0TA08642H

[48] Costa J Peixoto T Ferreira A Vaz F Lopes MA. Development and characterization of ZnO piezoelectric thin films on polymeric substrates for tissue repair. J Biomed Mater Res Part A (2019) 107(10):2150–9. doi: 10.1002/jbm.a.36725 31094062

[49] Zhu Z Gou X Liu L Xia T Wang J Zhang Y. Dynamically evolving piezoelectric nanocomposites for antibacterial and repair-promoting applications in infected wound healing. Acta Biomaterialia (2023) 157:566–77. doi: 10.1016/j.actbio.2022.11.061 36481503

[50] Pasquet J Chevalier Y Pelletier J Couval E Bouvier D Bolzinger M-A. The contribution of zinc ions to the antimicrobial activity of zinc oxide. Colloids Surfaces A: Physicochemical Eng Aspects (2014) 457:263–74. doi: 10.1016/j.colsurfa.2014.05.057

[51] Kaushik M Niranjan R Thangam R Madhan B Pandiyarasan V Ramachandran C. Investigations on the antimicrobial activity and wound healing potential of ZnO nanoparticles. Appl Surf Sci (2019) 479:1169–77. doi: 10.1016/j.apsusc.2019.02.189

